# Letter to the editor: Plenty of coronaviruses but no SARS-CoV-2

**DOI:** 10.2807/1560-7917.ES.2020.25.8.2000171

**Published:** 2020-02-27

**Authors:** Philippe Colson, Bernard La Scola, Vera Esteves-Vieira, Laetitia Ninove, Christine Zandotti, Marie-Thérèse Jimeno, Céline Gazin, Marielle Bedotto, Véronique Filosa, Audrey Giraud-Gatineau, Hervé Chaudet, Philippe Brouqui, Jean-Christophe Lagier, Didier Raoult

**Affiliations:** 1 Institut Hospitalo-Universitaire (IHU) Méditerranée Infection, Marseille, France; 2 Aix-Marseille University, Institut de Recherche pour le Développement (IRD), Assistance Publique - Hôpitaux de Marseille (AP-HM), Microbes Evolution Phylogeny and Infections (MEPHI), France; 3 Unité des Virus Emergents (UVE), Aix-Marseille University, IRD 190, Inserm 1207, IHU Méditerranée Infection, Marseille, France; 4 Service de l'Information Médicale, Hôpital de la Timone, Marseille, France; 5 Aix-Marseille University, Institut de Recherche pour le Développement (IRD), Assistance Publique - Hôpitaux de Marseille (AP-HM), Vecteurs – Infections Tropicales et Méditerranéennes (VITROME), Marseille, France; 6 French Armed Forces Center for Epidemiology and Public Health (CESPA), Service de Santé des Armées (SSA), Marseille, France

**Keywords:** Coronavirus, respiratory infections, SARS-CoV-2, France

**To the editor: **We read with interest the recent article by Reusken et al. about laboratory readiness for molecular testing of the novel coronavirus 2019, recently named severe acute respiratory syndrome coronavirus 2 (SARS-CoV-2) in expert laboratories in 30 European countries [1]. At the time of the Middle East respiratory syndrome (MERS)-coronavirus epidemic in 2012, we had highlighted the absence of diagnosis of this virus among travellers returning from the Hajj pilgrimage, which contrasted with the considerable anxiety relating to this emerging infection and its risk of importation and spread in mainland France [2]. Instead of MERS-CoV, influenza A and B viruses had been detected. This illustrated the major disconnect between the fear of a hypothetical spread in France of a virus emerging in the Middle East and the reality of the absence of diagnosed cases, while concomitantly the very real and high incidence of respiratory viruses common worldwide and in our country and their associated mortality appeared largely neglected. Seven years later, the emergence of SARS-CoV-2 in December 2019 reproduced this pattern of disproportionate fear of importation and spread of infections in mainland France while the cases reported worldwide remain almost only localised in China as only 34 people died of this disease (Covid-19) outside China as at 25 February 2020 [3].

In our reference institute for infectious diseases, we have been implementing since the end of January 2020 PCR detection of SARS-CoV-2 RNA using several systems, including those released at the European level [4]. In total, we have tested to date (as at 19 February 2020) 4,084 respiratory samples by PCR and all the tests have been negative for SARS-CoV-2. These tests were carried out on the samples of 32 suspected SARS-CoV-2 cases, 337 people repatriated at the beginning of February 2020 from China tested twice, 164 patients who died in public hospitals in Marseille between 2014 and 2019 of whom at least one respiratory sample had been sent to our laboratory, and they also included 3,214 respiratory samples sent since January 2020 to our laboratory to search for a viral aetiology. In striking contrast, we have tested 5,080 respiratory samples for various suspected respiratory viral infections since 1 January 2020 and identified in 3,380 cases respiratory viruses. In decreasing order of frequency, they were: influenza A virus (n = 794), influenza B virus (n = 588), rhinovirus (n = 567), respiratory syncytial virus (n = 361), adenovirus (n = 226), metapneumovirus (n = 192), enterovirus (n = 171), bocavirus (n = 83), parainfluenza virus (n = 24), and parechovirus (n = 8). Among the diagnosed viruses, there were also 373 common human coronaviruses (HCoV), including 205 HCoV-HKU1, 94 HCoV-NL63, 46 HCoV-OC43, and 28 HCoV-229E [5]. Furthermore, analysis of the mortality associated with these viruses has been able to show that since 1 January 2020, one patient died after being diagnosed with HCoV-HKU1, and respiratory viruses were found in 13 other patients who died, which included influenza A virus (3 cases), respiratory syncytial virus (3 cases), rhinovirus (5 cases), adenovirus (1 case) and metapneumovirus (1 case). Retrospectively, analysis of deaths in patients who have had a respiratory sample has shown that at least nine patients have died between 2017 and 2019 after being diagnosed with one of the four coronaviruses commonly circulating in humans [6].

Thus, it is surprising to see that all the attention focused on a virus whose mortality ultimately appears to be of the same order of magnitude as that of common coronaviruses or other respiratory viruses such as influenza or respiratory syncytial virus, while the four common HCoV diagnosed go unnoticed although their incidence is high. In fact, the four common HCoV are often not even identified in routine diagnosis in most laboratories, although they are genetically very different from each other [7] and associated with distinct symptomatology [8].

## References

[1] Reusken CBEM Broberg EK Haagmans B Meijer A Corman VM Papa A On Behalf Of Evd-LabNet And Erli-Net Laboratory readiness and response for novel coronavirus (2019-nCoV) in expert laboratories in 30 EU/EEA countries, January 2020. Euro Surveill. 2020;25(6).2000082. 10.2807/1560-7917.ES.2020.25.6.2000082 32046815 PMC7029448

[2] Raoult D Charrel R Gautret P Parola P From the Hajj: it’s the flu, idiot. Clin Microbiol Infect. 2014;20(1):O1. 10.1111/1469-0691.12448 24256052 PMC7129902

[3] World Health Organization (WHO). Coronavirus disease 2019 (COVID-19) Situation Report – 36. Geneva: WHO; 2020. Available from: https://www.who.int/docs/default-source/coronaviruse/situation-reports/20200225-sitrep-36-covid-19.pdf?sfvrsn=2791b4e0_2

[4] Corman VM Landt O Kaiser M Molenkamp R Meijer A Chu DK Detection of 2019 novel coronavirus (2019-nCoV) by real-time RT-PCR. Euro Surveill. 2020;25(3):10-7917. 10.2807/1560-7917.ES.2020.25.3.2000045 31992387 PMC6988269

[5] Surveillance microbiologique à l’AP-HM Point en semaine 7 (du 10 au 16 Février 2020). [Microbiological surveillance at the AP-HM. Update in week 7 (10 to 16 February 2020)]. Marseille: IHU Méditerranée Infection; 19/02/2020. French. Available from: https://www.mediterranee-infection.com/wp-content/uploads/2020/02/BEH-10-02-2020v.pdf

[6] Le « Global burden of infections » des hôpitaux publics de Marseille et de la région Provence-Alpes-Côte d’Azur. [The global burden of infections of the public hospitals in Marseille and the Region]. Marseille: IHU Méditerranée Infection; 19/02/2020. French. Available from: https://www.mediterranee-infection.com/le-global-burden-of-infections-des-hopitaux-publics-de-marseille-et-de-la-region-provence-alpes-cote-dazur/

[7] Cui J Li F Shi ZL Origin and evolution of pathogenic coronaviruses. Nat Rev Microbiol. 2019;17(3):181-92. 10.1038/s41579-018-0118-9 30531947 PMC7097006

[8] Zeng ZQ Chen DH Tan WP Qiu SY Xu D Liang HX Epidemiology and clinical characteristics of human coronaviruses OC43, 229E, NL63, and HKU1: a study of hospitalized children with acute respiratory tract infection in Guangzhou, China. Eur J Clin Microbiol Infect Dis. 2018;37(2):363-9. 10.1007/s10096-017-3144-z 29214503 PMC5780525

